# Potential Therapeutic Effects of Mi-Jian-Chang-Pu Decoction on Neurochemical and Metabolic Changes of Cerebral Ischemia-Reperfusion Injury in Rats

**DOI:** 10.1155/2022/7319563

**Published:** 2022-05-06

**Authors:** Jingjing Liu, Lingling Yang, Yang Niu, Chao Su, Yingli Wang, Ruru Ren, Jianyu Chen, Xueqin Ma

**Affiliations:** ^1^Department of Pharmaceutical Analysis, School of Pharmacy; Key Laboratory of Hui Ethnic Medicine Modernization, Ministry of Education, Ningxia Medical University, 1160 Shenli Street, Yinchuan 750004, China; ^2^School of Pharmacy, Lanzhou University, Lanzhou 730000, China; ^3^Fujian University of Traditional Chinese Medicine, No. 1, Huatuo Road, Minhoushangjie, Fuzhou 350122, China

## Abstract

As a traditional Chinese medicine formula, Mi-Jian-Chang-Pu decoction (MJCPD) has been successfully used in patients with language dysfunction and hemiplegia after ischemic stroke (IS). Given the excellent protective effects of MJCPD against nerve damage caused by IS in clinical settings, the present investigation mainly focused on its underlying mechanism on ischemia-reperfusion (IR) injury. Firstly, by applying the MCAO-induced cerebral IR injury rats, the efficacy of MJCPD on IS was estimated using the neurological deficit score, TTC, HE, and IHC staining, and neurochemical measurements. Secondly, an UHPLC-QTOF-MS/MS-based nontargeted metabolomics was developed to elucidate the characteristic metabolites. MJCPD groups showed significant improvements in the neurological score, infarction volume, and histomorphology, and the changes of GSH, GSSG, GSH-PX, GSSG/GSH, LDH, L-LA, IL-6, TNF-*α*, and VEGF-c were also reversed to normal levels after the intervention compared to the MCAO model group. Metabolomics profiling identified 21 different metabolites in the model group *vs.* the sham group, 10 of which were significantly recovered after treatment of MJCPD, and those 10 metabolites were all related to the oxidative stress process including glucose, fatty acid, amino acid, glutamine, and phospholipid metabolisms. Therefore, MJCPD might protect against IS by inhibiting oxidative stress during IR.

## 1. Introduction

Cerebral stroke, including hemorrhagic stroke (HS) and ischemic stroke (IS), is a type of acute cerebrovascular disease characterized by high mortality, high incidence, and high disability rates [1]. It is recorded that approximately 87% of cerebral stroke is caused by ischemia [2], which might lead to disturbance of consciousness and limb paralysis in severe cases. Cerebral ischemia-reperfusion (IR) is a way of recanalization of the occluded vessels after ischemia, which could restore brain tissue blood supply timely but aggravate brain tissue damage further [3]. Numerous literatures have shown that IR might cause nerve cell apoptosis and necrosis, and the main mechanisms were believed to be related to ischemia and hypoxia [4]. Specifically, restoration of blood flow after reperfusion could cause intracellular calcium overload, excessive formation of oxygen free radicals, cytochrome C and inflammatory mediators release, overactivation of cytokines, and leukocyte adhesion appendage increase, then trigger “no-reflow phenomenon” through the toxicity of excitatory amino acid, inflammatory response, and microvascular occlusion [5, 6], further affect the cerebral microcirculation and cellular microenvironment, and ultimately led to the death of neuronal cells in the ischemic penumbra. Therefore, multiple factors and pathways could exacerbate brain damage [7]. Clinically, we also found that when IS patients received thrombolytic drugs to make vascular recanalization, the neurological function improved at once, but more severe cerebral edema or intracranial hemorrhage occurred simultaneously, leading to worsening symptoms and even death [8]. In general, once cerebrovascular obstruction was reopened, and even blood flow was restored, a series of biochemical cascades occurred in the restoration of blood flow, which in turn aggravated brain damage including IR injury [9]. At present, the main methods for clinical treatment of IR were either thrombolytic therapy or surgery, and the only therapeutic drug that has been approved by Food and Drug Administration was the tissue-type plasminogen activator (t-PA) [10, 11]. However, due to the t-PA treatment window being only 4.5 hours, this results in less than 5% of patients that could be effectively treated [12]. Based on the mechanisms of brain injury in acute cerebral ischemia which have not yet been fully clarified till now, the development of therapeutic agents was thus limited.

To date, traditional Chinese medicine (TCM), including extract and isolated natural ingredients, has received more attention. TCM could protect against and treat dozens of complicated diseases by acting through multiple pathways and targets and have achieved some success in reducing the brain injury caused by cerebral IR [13]. According to the theory of TCM, “promoting blood circulation, removing stasis, clearing away heat, and removing toxin” have been widely recognized as effective methods for the clinical prevention and treatment of IS. Blood-activating and stasis-resolving agents played an important role in improving the circulation of the brain, protecting the nerve cells and removing the free radicals, whereas heat-clearing and toxicity-removing agents focused on alleviating the inflammatory response, activating the self-protection mechanism of brain cells, and reducing the secondary brain injury due to IS. The synergy of the above different bioactive agents has made TCM for the prevention and treatment of cerebral IR injury possible [13]. At present, some TCMs, such as *Curcuma longa* L., *Rabdosia rubescens* (Hemsl.) Hara, *Ilex pubescens* Hook. et Arn., *Leonurus japonicus* Houtt, *Acorus tatarinowii* Schott, and *Crocus sativus* L., which possessed the function of clearing heat, detoxifying, activating blood, and removing blood stasis, were proven to be effective on resisting cerebral ischemic injury [14, 15].

Two thousand years ago, the people of Chinese ethnic minorities found Mi-Jian-Chang-Pu decoction (MJCPD), composed of *Crocus sativus* L. and *Acorus tatarinowii* Schott, which could improve the language dysfunction and hemiplegia of IS patients. The above description was recorded in the classical medicinal book named *Huihui Prescription* [16]. Currently, the therapeutic effects of MJCPD on stroke-related lipid metabolic disorders have been validated in clinical studies [17]. Besides MJCPD itself, *C. sativus*, one of the compositions of MJCPD, was also proven to possess neuroprotective effect. *C. sativus* could activate blood circulation and remove blood stasis, which was believed to be related to antioxidant and anti-inflammatory mechanisms [18], and its aqueous extract could improve behavioral, biochemical, and neurotransmitter deficits, as well as histological change caused by IR [19]. In relative terms, *A. tatarinowii* is the other composition of MJCPD; little was known about the effects of its extract on the central nervous system diseases including cerebral ischemia. However, *β*-asarone, a major component of *A. tatarinowii*, could permeate the blood brain-barrier [20] and then reduce neuronal apoptosis in the rat hippocampus and attenuate the injuries of the blood vessel endothelium and nerve cells of the cortex [21, 22]. Thus, we hypothesize that MJCPD possesses protective effect on ischemia-induced brain infarction. In the present study, the neuroprotective effects and the underlying mechanisms of MJCPD on neurobehavioral functions were estimated. Besides a pharmacodynamics approach, a metabolomics technique based on UHPLC-QTOF-MS/MS was also applied to investigate the serum metabolite profiling of cerebral ischemic rats induced by middle cerebral artery occlusion (MCAO), as well as the effect of MJCPD and its action mechanism against IS. Accordingly, the potential biomarkers related to cerebral IR were identified, and their metabolic pathways were further discussed.

## 2. Materials and Methods

### 2.1. Plant Materials, Chemicals and Solvents, *Crocus sativus* L., and *Acorus tatarinowii*

Schott was purchased from Ming De Chinese Herbal Medicine Company (Ningxia, China) and identified by Professor Lin Dong, Department of Pharmacognosy, Ningxia Medical University. Voucher specimens (20150015#, 20150016#) were conserved at the herbarium of School of Pharmacy, Ningxia Medical University. Standard reference of crocin I (11158-201303, 92.6%) was obtained from the National Institutes for Food and Drug Control. Crocin II (AF7121712, ≥98%), *α*-asarone (CAS 2883-989, ≥98%), and *β*-asarone (CAS5273-86-9, ≥98%) were purchased from Chengdu Efa Bio-Technology Co., Ltd. (Chengdu, China). Butylphthalide (0.1 g/pill) was obtained from Enbipu Pharmaceutical Co., Ltd. Triphenyltetrazolium chloride (TTC) was purchased from Beijing Solarbio Science Technology Co., Ltd. (Beijing, China). Nitric oxide (NO), lactate dehydrogenase (LDH), L-lactic acid (L-LA), malondialdehyde (MDA), glutathione (GSH), glutathione disulfide (GSSG), and glutathione peroxidase (GSH-PX) assay kits were provided from Nanjing Jiancheng Biotechnology Co., Ltd. (Nanjing, China). Tumor necrosis factor-*α* (TNF-*α*), vascular endothelial growth factor-c (VEGF-C), interleukin-10 (IL-10), and IL-6 ELISA kits were purchased from Elabscience Biotechnology Co., Ltd. (Wuhan, China). VEGF, caspase-3, and GFAP antibodies were purchased from Abcam Bio Co., Ltd. Acetonitrile and methanol (HPLC grade) were obtained from Fisher Scientific (MA, USA). Formic acid (HPLC grade) was purchased from Merck Scientific (MA, USA). All the other chemicals used were of analytical grade.

### 2.2. Preparation of the MJCPD Sample

20.0 g of air-dried stems of *A. tatarinowii* was extracted by using a steam distillation method for 3 hours; then, the oil was isolated, and the residue was reextracted with water (200 mL, 2 × 2 h) for 2 times. The filtrates were combined, dried, evaporated, and mixed with the oil to obtain the crude extracts of *A. tatarinowii* (4.6 g). *A. tatarinowii* extracts were then mixed with the powders of *C. sativus* (20.0 g, smashed and sieved using a 100-mesh sieve) and dissolved in 0.5% CMC-Na to get the MJCPD sample.

### 2.3. HPLC Analysis of the MJCPD Sample

We elucidated the major constituents of MJCPD by using HPLC with the corresponding standard references. The chromatography conditions were described as follows: TSK-GEL C18 column (4.6 mm × 250 mm, 5 *μ*m); the binary mobile phase consists of water with 0.1% phosphoric acid (mobile phase A) and 60% methanol (mobile phase B), using a gradient elution as follows: 0~5 min, 40%~60% B; 5~20 min, 60%~90% B; 20~21 min, 90%~40% B; and 21~23 min, 40% B. The column temperature was held at ambient temperature, with the flow rate being 1.0 mL/min, detection wavelength being 280 nm, and the sample injection volume being 10 *μ*L.

### 2.4. Rat Model and Administration

90 male Sprague-Dawley rats (280-320 g) were purchased from Ningxia Medical University (animal license number: SCXK-2015-0001; Ningxia, China), and every 3 rats were kept in one cage, maintained in a well-ventilated lab at constant ambient temperature (24.0 ± 0.5°C) and air humidity (45-50%) with a 12 h light/12 h dark cycle. All animals were allowed food and water ad libitum throughout the study. Before the surgery, animals were acclimatized for 7 days; then, they were randomly divided into 6 groups: those intragastrically administered with butylphthalide (120 mg/kg/day) were the positive control (NBP); those administered with 115, 230, and 460 mg/kg/day of MJCPD were low (MJCPDL), moderate (MJCPDM), and high (MJCPDH) dosage groups, respectively; and those administered with vehicle (0.5% CMC-Na) were model (IR) and control (SHAM) groups, respectively. There were 15 SD rats per group. The rats were fasted for 12 h before surgery but were allowed free access to water.

The MCAO operations were produced using the intraluminal filament model by the method of Longa et al. [23]. Briefly, all the rats were anesthetized with 1% pentobarbital sodium through intraperitoneal injection; then, the right common carotid artery (CCA), external carotid (ECA), and internal carotid artery (ICA) were exposed and isolated from connective tissues, respectively. To ligate the CCA proximal portion and ECA bifurcation, a poly-L-lysine-coated nylon monofilament (2432-A4) was inserted into the ICA through the ECA and advanced 18 mm to 20 mm as the distance from the bifurcation according to the weight of the animal until it blocked the origin of the right middle cerebral artery. After occlusion for 2 h, the suture was removed lightly, reperfusion was realized, and a MCAO reperfusion model was built. In the sham-operated group, the same surgical operations were performed except the arteries were not occluded. The room temperature was maintained at 25 ± 2°C, and the rectal temperature of the animal was continuously kept at 37.0 ± 0.5°C using a rat thermostat bench (Beijing Xinong Technology Co., Ltd.) during the entire operation. Animal protection and use and all procedures were in accordance with the requirements of the Animal Ethics Committee (NXMU2018-192).

### 2.5. Neurological Deficit Measurements

After 24 h of IR, the neurological functions of all the rats were evaluated according to the Zea Longa test scores, and the tests must be completed within 5 minutes. The scoring standard was as follows: score 0, no neurological deficit; score 1, a slight decrease in forelimb flexion; score 2, severe decrease in forelimb flexion and rotating like a contralateral rotation at the end of the lift; score 3, spontaneous rotation or contralateral rotation to the affected side; score 4, consciousness reduced and unable or difficult to move; and score 5, no response to stimulation.

### 2.6. Estimation of Infarct Volume by TTC Staining

One-third of rats in each group were anesthetized and killed after the neurological deficit score measurements; the brain tissues were taken out rapidly and cooled at -20°C immediately for 15 minutes. Then, each was dissected into 2 mm thick slices and placed in 1.5% TTC solution, and incubation was performed for 30min at 37°C without light. The tissue pieces were carefully turned over to ensure uniform staining during the process, and the white part represented infarct whereas the red implied normal. Photographs were taken for the image analysis.

### 2.7. Histology and Immunohistochemistry

Another one-third of rats in each group were processed for histology and immunohistochemistry. Sampling procedures were as follows: a perfusion needle was inserted into the left ventricle, and the right auricle was cut open. The samples were perfused with 0.9% physiological saline for 15 min and 4% precooled paraformaldehyde for 15 min. All rats were decapitated and the brain tissues were separated on ice, and a 4 mm coronal section between the root of the optic chiasma and the corpora quadrigemina was fixed in 4% paraformaldehyde for 24 h. Then, the fixed tissue was dehydrated, rendered transparent, dipped in wax, and embedded to prepare conventional paraffin sections, which were stained with HE for the histological evaluation. The other coronal sections after antigen retrieval with sodium citrate buffer solution (pH 6.0) were immunostained with rabbit anti-VEGF (1 : 75), anti-caspase-3 (1 : 50), and anti-GFAP (1 : 1000), respectively, followed by incubation with goat anti-rabbit IgG conjugated to a peroxidase-labeled dextran polymer at 37°C for 1 h. After rinsing with PBS, the immune reactivity was visualized with the DAB detection kit. The positive ratios were quantitatively measured in coronal sections through IPP 6.0 software, and the mean value was used for analysis.

### 2.8. Measurements of Cytokines in Brain Tissues

Again, the brain tissues of the remaining one-third of rats were taken out and washed with precold PBS buffer (0.01 M, pH 7.4) and homogenized at 5000 g for 10 min at 4°C. The supernatants were collected for the detection of VEGF-c, IL-6, IL-10, and TNF-*α* with corresponding ELISA kits. All procedures were performed according to the manufacturer's instructions.

### 2.9. Measurement of Oxidative Stress-Related Biochemical Indicators as Well as the Levels of LDH and L-LA

After 24 h of reperfusion, the rats were sacrificed and the serum was obtained from the whole blood with centrifugation at 4000 g for 15 min at 4°C. The samples were collected and stored at -80°C for the subsequent biochemical factor determination and metabolomics experiments. A portion of the serum of each rat was used to determine the levels of NO, MDA, GSH, GSSG, and GSH-PX as well as LDH and L-LA with relevant kits. All procedures were performed according to the manufacturers' instructions.

### 2.10. Metabolomics Analysis

#### 2.10.1. Sample Collection and Preparation

The serum supernatant of each rat was mixed with acetonitrile at a ratio of 1 : 3 (serum supernatant: acetonitrile, *v*/*v*) to deposit the protein. After being vortexed for 30 s, the resulting solutions were centrifuged (13000 rpm, 10 min) at 4°C. Finally, 1 *μ*L of the supernatant was injected to UHPLC-QTOF-MS/MS for metabolomics analysis.

#### 2.10.2. Analysis Conditions

The metabolomics measurement was performed using an Agilent 1290 Infinity UHPLC system combined with a 6545 quadrupole time-of-flight mass spectrometer (Agilent Technologies, Santa Clara, CA, USA) rigged with an electrospray interface. Chromatographic separation was conducted on the Agilent EclipsePlus C18 column (1.8 *μ*m, 2.1 × 50 mm) with a flow rate of 0.25 mL/min at a temperature of 40°C. The mobile phase contained 0.1% formic acid in water (A) and 0.1% formic acid in acetonitrile (B); gradient elution procedures were as follows: 0-1 min, 1%-20% B; 1-10 min, 20%-70% B; 10-14 min, 70%-82% B; 14-15 min, 82%-85% B; 15-16 min, 85%-99% B; 16-17.5 min, 99% B; 17.5-18.5 min, 99%-1% B; and 18.5-20 min, 1% B. The injection volume was 1.5 *μ*L. ESI-MS/MS conditions were as follows: capillary voltage 3500 V, nebulizer pressure 40 psig, flow rate of drying gas 12 L/min, temperature 350°C, and scanning *m*/*z* range 100-1000.

#### 2.10.3. Method Validation

The precision, repeatability, stability, and matrix effects as well as the recovery of extraction were used to validate the method. Meanwhile, the system stability was carried out by injecting a quality control (QC) sample every 6 samples during the whole sample analysis.

#### 2.10.4. Data Processing

All samples were analyzed according to the above conditions. The UHPLC-QTOF-MS/MS data of all determined samples were firstly converted into the “mz. data” file format using MarkerView 1.2 software, and the abundance of aligned features was normalized using an internal standard (astragaloside A). Then, the data were imported into the XCMS software package for peak identification, filtering, and alignment, and the three-dimensional data matrix, including names, retention times, *m*/*z* pairs, and normalized ion intensities, were further exported to the SIMCA-P 14.1 software package for principal component analysis (PCA) and partial least squares discriminant analysis (PLS-DA). Meanwhile, the univariate analysis *t*-test was also performed. Metabolite peaks with VIP value greater than 1 and *p* value less than 0.05 were considered significant differential biomarkers. Finally, the pathway analysis of these differential metabolites was performed using HMDB, METLIN, and KEGG databases.

### 2.11. Statistical Analysis

All values were expressed as mean ± SD. The data were analyzed by using the SPSS 23.0 software with one-way analysis of variance followed by the LSD *t*-test. Only those with *p* < 0.05 were considered statistically significant.

## 3. Results

### 3.1. Main Chemical Constituents of MJCPD

**Figure 1:**
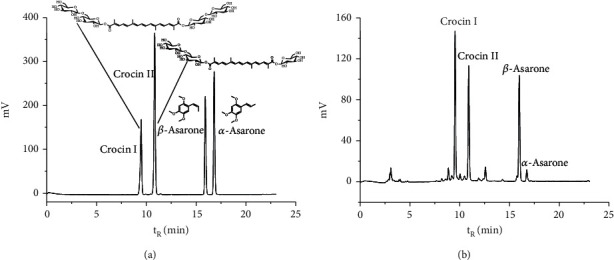
HPLC analysis of the MJCPD extract: (a) chromatography of standard references and their chemical structures; (b) chromatography of the MJCPD sample. The main constituents were crocin I, crocin II, *α*-asarone, and *β*-asarone.

By using standard references, four main constituents including crocin I, crocin II, *α*-asarone, and *β*-asarone were identified in the MJCPD extract, as shown in Figure 1.

### 3.2. Neurobehavioral Scores and Cerebral Infarction Evaluation

**Figure 2:**
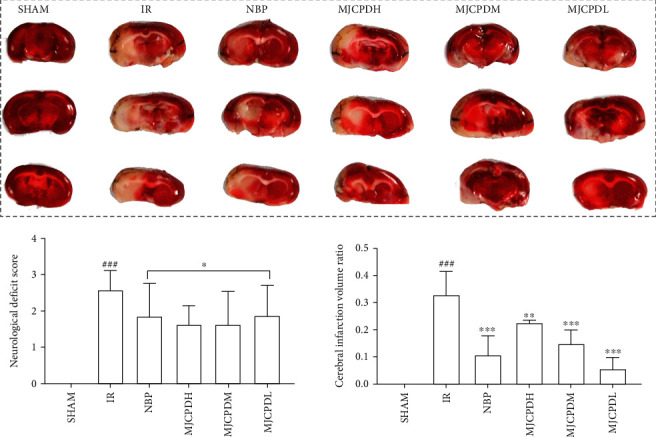
Protective effects of MJCPD on the morphology of cerebral tissue observed by TTC staining, neurological defects, and infract volume. Data were expressed as mean ± SD. ^###^*p* < 0.001 as compared to SHAM group; ^∗^*p* < 0.05, ^∗∗^*p* < 0.01, and ^∗∗∗^*p* < 0.001 as compared to the IR group.

The neurological deficit score (Figure 2) of rats in the IR model group was significantly higher (*p* < 0.001) than that of the SHAM group, which indicated that the cerebral IR model was established successfully. After being treated with MJCPD (115, 230, and 460 mg/kg/day) and NBP, respectively, the neurological scores of rats were apparently decreased (*p* < 0.05), as compared with those of the rats of the IR model group.

At 24 h after IR, the TTC staining was performed and the infarct volume was estimated. As shown in Figure 2, no infarction was observed in rats of the SHAM group, whereas a significant increased infarction volume was observed in those of the IR model group (*p* < 0.001). Both the MJCPD- (115, 230, and 460 mg/kg/day) and NBP-treated groups displayed markedly decreased infarction volume (*p* < 0.01 and *p* < 0.001) as compared with the IR model group, indicating that MJCPD had significant therapeutic effects on nerve and cerebral injuries.

### 3.3. Histopathological and Immunohistochemistry Examination

**Figure 3:**
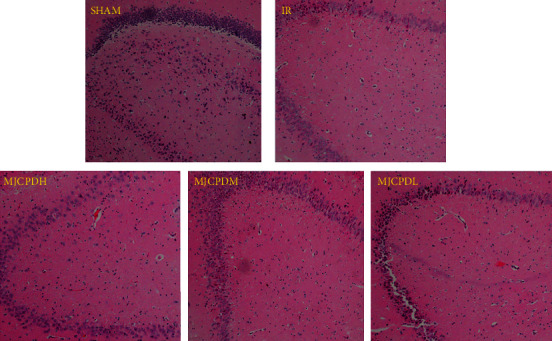
Histopathological results of brain tissues in SHAM, IR, and MJCPD-treated groups (original magnification: 4x).

**Figure 4:**
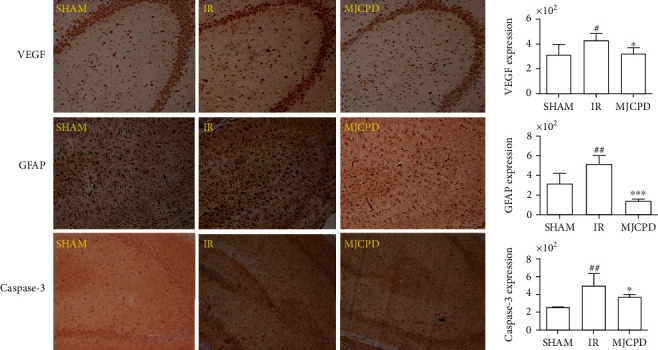
Immunohistochemistry results of brain tissues in SHAM, IR, and MJCPD-treated groups (original magnification: 4x). Data were expressed as mean ± SD. ^#^*p* < 0.05 and ^##^*p* < 0.01 as compared to the SHAM group; ^∗^*p* < 0.05 and ^∗∗∗^*p* < 0.001 as compared to the IR group.

The histological properties were observed by HE staining of brain tissue; the results are shown in Figure 3. In the IR model group, the brain tissue presented obviously damaged neurons and a shrunken nucleus. After being treated with MJCPD (230 mg/kg/day), the abnormality histological characteristics were significantly ameliorated and the degree of improvement was similar to that of the SHAM operation group. To further explore whether MJCPD could modulate neuronal cell death or not, VEGF, GFAP, and caspase-3 were detected using immunohistochemical analysis. As shown in Figure 4, the quantities of VEGF, GFAP, and caspase-3 in the MJCPD-treated groups were dramatically reduced (*p* < 0.05, *p* < 0.001, and *p* < 0.05, respectively) as compared to the IR model group. The results demonstrated that MJCPD could improve neuroinflammation and suppress cell death in IR rats.

### 3.4. Effects of MJCPD on the Levels of NO, MDA, GSH, and GSSG and the Activity of GSH-PX

**Figure 5:**
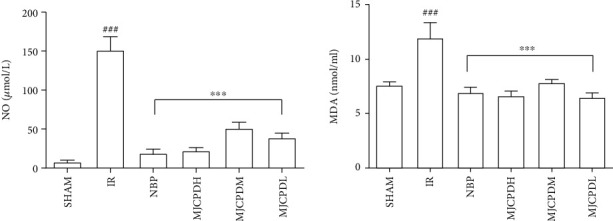
Effects of MJCPD on NO and MDA levels in serum of rats with cerebral IR injuries. Data were expressed as mean ± SD. ^###^*p* < 0.001 as compared to the SHAM group; ^∗∗∗^*p* < 0.001 as compared to the IR group.

**Figure 6:**
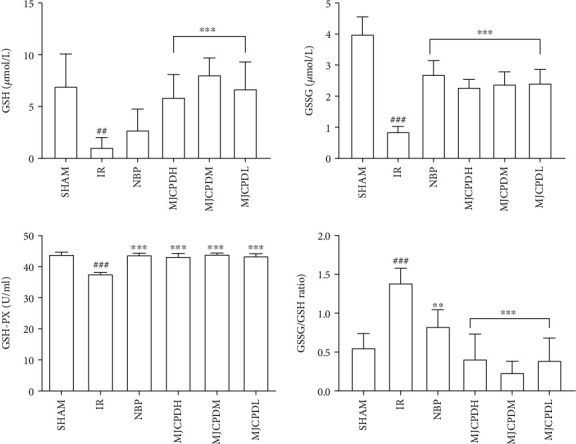
Effects of MJCPD on GSH, GSH-PX, and GSSG levels in serum of rats with cerebral IR injuries. Data were expressed as mean ± SD; ^##^*p* < 0.001 and ^###^*p* < 0.001 as compared to the SHAM group; ^∗∗^*p* < 0.01 and ^∗∗∗^*p* < 0.001 as compared to the IR group.

In order to explore the effects of MJCPD on antioxidant activities, the oxidative stress markers including NO and MDA were estimated. As shown in Figure 5, the contents of NO and MDA were significantly higher in rats of the IR model group (*p* < 0.001) as compared with the SHAM rats, whereas MJCPD- (115, 230, and 460 mg/kg/day) and NBP-treated groups showed significantly lower levels of NO and MDA (*p* < 0.001) as compared to the IR model group. Meanwhile, Figure 6 shows significantly reduced levels of GSH, GSSG, and GSH-PX and increased GSSG/GSH ratio in the rats of the IR group (*p* < 0.01) as compared to the SHAM rats. MJCPD and NBP treatment could significantly reverse the changes of GSH, GSSG, GSH-PX, and GSSG/GSH, which was almost close to the level of SHAM rats. The results implied that MJCPD played an important role in recovering cerebral damage in IR rats.

### 3.5. Effects of MJCPD on the Levels of LDH and L-LA

**Figure 7:**
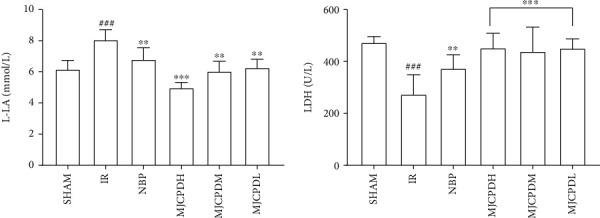
Effects of MJCPD on L-LA and LDH activities in serum of rats with cerebral IR injuries. Data were expressed as mean ± SD. ^###^*p* < 0.001 as compared to the SHAM group; ^∗∗^*p* < 0.01 and ^∗∗∗^*p* < 0.001 as compared to the IR group.

As shown in Figure 7, the content of L-LA in the rats of the IR model group was significantly higher (*p* < 0.001), whereas the level of LDH was markedly lower (*p* < 0.001) than that of the SHAM rats. Also, the levels of L-LA and LDH in MJCPD treatment groups were obviously reversed (*p* < 0.001 and *p* < 0.01, respectively) as compared to those in the IR model group. The results showed that MJCPD could alleviate oxidative injury in IR rats.

### 3.6. Measurements of Cytokines in Brain Tissues

**Figure 8:**
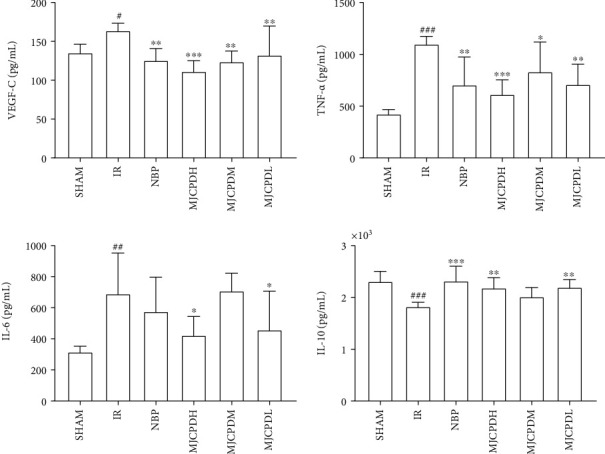
Effects of MJCPD on VEGF-c, TNF-*α*, IL-6, and IL-10 activities in brain tissues of rats with cerebral IR injuries. Data were expressed as mean ± SD. ^#^*p* < 0.05, ^##^*p* < 0.01, and ^###^*p* < 0.001 as compared to the SHAM group; ^∗^*p* < 0.05, ^∗∗^*p* < 0.01, and ^∗∗∗^*p* < 0.001 as compared to the IR group.

The effects of MJCPD on inflammatory reactions were explored by determining the content of inflammatory cytokine in the brain tissue which is shown in Figure 8. In the IR injury group, the expression of IL-10 was obviously decreased (*p* < 0.001), while IL-6, TNF-*α*, and VEGF-c were dramatically increased (*p* < 0.05, *p* < 0.01, and *p* < 0.001, respectively) as compared to that in the SHAM group. Expectedly, the levels of IL-6 and IL-10 were markedly reversed in the MJCPD high- and low-dose groups, and the levels of VEGF-c and TNF-*α* in MJCPD-treated groups were significantly decreased as compared to those in the IR model group. These results implied that MJCPD could adjust the inflammatory response of IR rats back to normal levels.

### 3.7. Metabolomics Study

#### 3.7.1. Methodological Findings and System Stability

By employing QC samples, the optimization of separation and determination conditions of UHPLC-QTOF-MS/MS was established. Data acquisition in positive ion mode indicated that the QC samples showed good repeatability (Supplementary Figure 1), which implied the stability of the instrument during the data acquisition. Meanwhile, the representative total ion chromatograms of SHAM and IR groups, as shown in Supplementary Figure 2, indicated that the peaks were of good shape and separated from each other. The above results showed that the analysis method was suitable for the subsequent sample study.

As shown in Table 1, the results of matrix effect (92.19%~117.09%) and extraction recovery (86.56%~113.27%) showed that the sample processing method was suitable for the study. At the same time, the RSD of the peak areas for precision and stability were 1.69%~13.58% and 1.27%~14.15% in positive ion mode, respectively, which indicated that our established method was reliable and the analysis was satisfied.

#### 3.7.2. Data Acquisition and Processing of the Metabolite Profile

**Figure 9:**
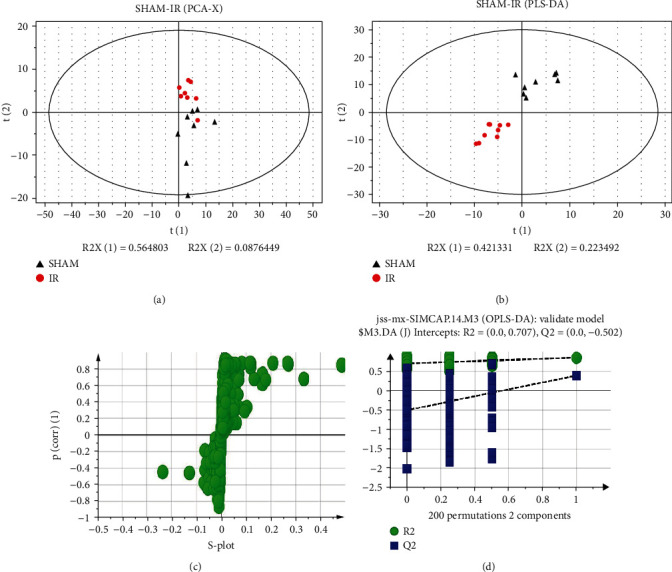
Multivariate statistical analysis of the SHAM group and IR group in positive ion mode: (a) PCA-X score plot of the SHAM group and IR group, showing no obvious separation trend; (b) PLS-DA score plot of the SHAM group and IR group, showing a good separation trend; (c) scatter plot in the positive mode; (d) permutation test of the SHAM group and the IR group in the positive mode, showing the model fit was good.

Firstly, we applied PCA and PLS-DA score plots to explain distinct separation between the SHAM and IR model groups. As shown in Figure 9(a), the PCA score plots of positive ion mode showed a slightly significant difference between the SHAM and IR model groups. Then, by using PLS-DA, there was a significant separation trend between the two groups (Figures 9(b) and 9(c)). Meanwhile, the validated model (permutation test, 200 times) (Figure 9(d)) showed that the PLS-DA model was valid.

In our present experiments, the metabolite with VIP > 1 and *p* < 0.05 was regarded as a potential differential biomarker. Then, a total of 21 differentially endogenous metabolites were screened by using the databases of METIN, HMDB, and MetaboAnalyst and literatures based on the *m*/*z* of MS/MS fragmentation data and retention time; they were L-isoleucine, L-phenylalanine, PC (14 : 0/0 : 0), D-phenylalanine, 5-formylsalicylic acid, 1-heptadecanoyl-sn-glycero-3-phosphocholine, PC (16 : 0/0 : 0), 3-methylindole, glycerol 1-hexadecanoate, L-tryptophan, L-tyrosine, cytosine, 1-oleoyl-sn-glycero-3-phosphocholine, DL-proline, docosahexaenoic acid, 1-stearoyl-sn-glycerol-3-phosphocholine, D-leucine, DL-a-hydroxyvaleric acid, N-acetyl cysteine, pyroglutamic acid, and acetoacetate. The relative changes of differential metabolites in the SHAM, IR, and MJCPD groups are presented in Table 2.

#### 3.7.3. Metabolic Pathway Enrichment Analysis

**Figure 10:**
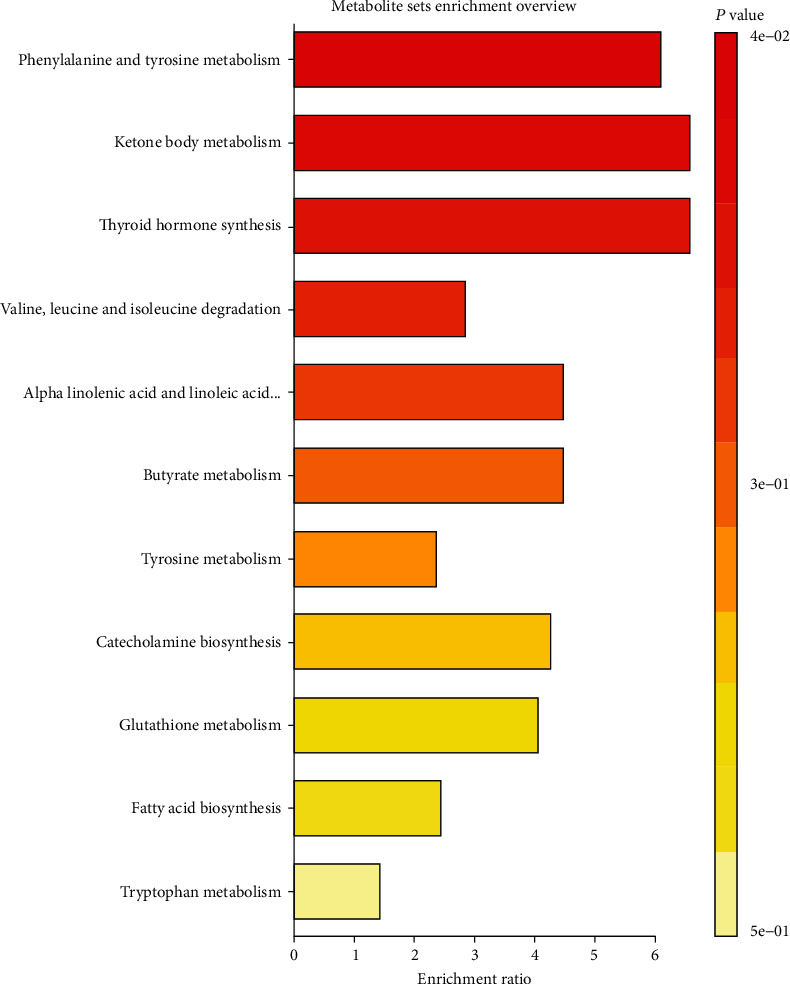
The metabolic pathways related to cerebral IR, as analyzed by MetaboAnalyst.

Based on the enrichment of 21 metabolites, as shown in Figure 10, a set of metabolism pathways were constructed to elaborate the biological significance of cerebral IR, successively; they were phenylalanine and tyrosine metabolism, ketone body metabolism, thyroid hormone synthesis, valine, leucine, and isoleucine degradation, alpha linolenic acid and linoleic acid metabolism, butyrate metabolism, tyrosine metabolism, catecholamine biosynthesis, glutathione metabolism, fatty acid biosynthesis, and tryptophan metabolism.

## 4. Discussion

Nowadays, cerebral ischemic injury still carries a high mortality rate globally regardless of therapeutic advances. Given the complex physiological and pathological mechanisms, a set of multifactorial processes including overformation of free radicals, overloaded intracellular calcium, excitotoxicity of amino acids, inflammatory reactions, enhanced anaerobic metabolism, and abnormal protein synthesis usually occurred, and consequently, neurological impairment, learning and memory disability, and cerebral infarct happened [24]. The cellular and subcellular lesions in cerebral ischemic injury may lead to injury of neurons and death of necrosis, apoptosis, pyroptosis, and autophagy [25]. At present, treatment of IS may involve administration of intravenous thrombolytic drugs such as tPA and conventional adjuvant therapies including anti-inflammatory, antioxidant, free radical scavenging, and neuroprotective drugs. However, it is like a double-edged sword which might make the cerebral ischemic injury more seriously. Therefore, other effective treatments including natural medicine remain urgently needed for the treatment of IS.

In this study, 21 metabolites and the corresponding metabolic pathways were identified in rats of the IR model group as compared to SHAM rats. Studies have shown that IS could cause central nervous system damage through oxidative stress, neuroinflammation, and other ways, which consequently caused many sequelae and complications such as cognitive impairment and gastrointestinal bleeding. In those processes, the imbalance of metabolic homeostasis between the brain and whole body had also been proven to play an important role. Accumulation of free radicals during the cerebral ischemia usually resulted in neurotoxicity by acting on polyunsaturated fatty acids, then leading to lipid peroxidation and triggering the cross-linking of macromolecules [26].

### 4.1. Glutathione Metabolism and Oxidative Stress

In our study, an improved MCAO rat model was used to mimic the cerebral IR and to estimate the anti-ischemic effect of MJCPD on IR rats. TTC and HE staining showed that MJCPD could exert a neuroprotection effect on cerebral IR injury in model rats. Oxidative stress was a state of imbalance between oxidation and antioxidant [27]; it could destroy cell macromolecules, resulting in infiltration of inflammatory neutrophils, as well as increased protease secretion, lipid peroxidation, and DNA damage. GSH, GSSG, GSH-PX, MDA, and LDH were generally used to measure and monitor the oxidative stress. GSH was proven to play a vital role in retaining cell biological function, and nerve cells were no exception. Under oxidative stress conditions, decreased GSH was the most common scavenger of reactive oxygen species, and its ratio with GSSG was regarded as a marker of oxidative stress [28]. In our experiments, the increased GSH, GSSG, and GSH-PX levels as well as the decreased GSSG/GSH ratio in the MJCPD-treated IR group implied that the protective mechanisms of MJCPD might be linked with its antioxidative property.

### 4.2. Inflammatory Response during the Cerebral Ischemia

IR inflammatory cytokines are important mediators of inflammatory responses in central nervous system diseases [29]; understanding the effects of inflammation on the IR might provide a new direction for the treatment of IS. Cerebral ischemia can induce the expression of adhesion molecules in endothelial cells and leukocytes and produce a large number of proinflammatory cytokines, which lead to the adhesion of white blood cells and extravasation into brain soft tissue [30]. IL-6, IL-10, and TNF-*α* were involved in neuronal apoptosis and the mediation of inflammatory cytokines during cerebral IR [31]. Meantime, VEGF is a hypoxia-inducible angiogenic peptide which was found to be associated with inflammatory cells and played an important role in the chronic neuroinflammation [32]. GFAP is a structural protein that is almost exclusively expressed in astrocytes and involved in glial plasticity and neuroinflammatory process in response to emotional experience, and its level can be used to assess neurologic deficits and monitor therapy of IS [33]. In our study, the immunohistochemical results were consistent with those reported in the literature and confirmed the above-mentioned view. Caspase-3 is a frequently activated death protease, which was a crucial mediator of programmed cell death [34]. In this study, MJCPD increased the levels of IL-10 but decreased the expression of IL-6 and TNF-*α*, as well as reducing the VEGF, GAFP, and caspase-3 expression; thus, we supposed that MJCPD could improve inflammatory response and inhibit cell apoptosis in rats with IR.

### 4.3. Phospholipid Metabolism

Phospholipid is an extremely important biomolecule in the brain. It is the main component of cell membrane and an important energy pool, which plays an important role in cell signal transduction. Lysophospholipid, an intermediate product of phospholipid metabolism, was believed to be closely related to oxidative stress injury [35]. Previous studies have shown that lysophosphatidylcholine could aggravate IR injury in nerve cells through increasing intracellular calcium under ischemic conditions. Lysophosphatidylcholines and lysophosphatidylethanolamines were proven to have neuroprotective effects [36]. In our study, the contents of lysoPC (14 : 0/0 : 0) and 1-oleoyl-sn-glycero-3-phosphocholine were significantly increased in MJCPD intervention groups, while the levels of lysoPC (16 : 0/0 : 0), 1-stearoyl-sn-glycerol-3-phosphocholine, and 1-heptadecanoyl-sn-glycero-3-phosphocholine were obviously decreased, which coincided with the published results [37] and also indicated that phospholipids and their metabolites were activated after IR.

### 4.4. Amino Acid Metabolism

**Figure 11:**
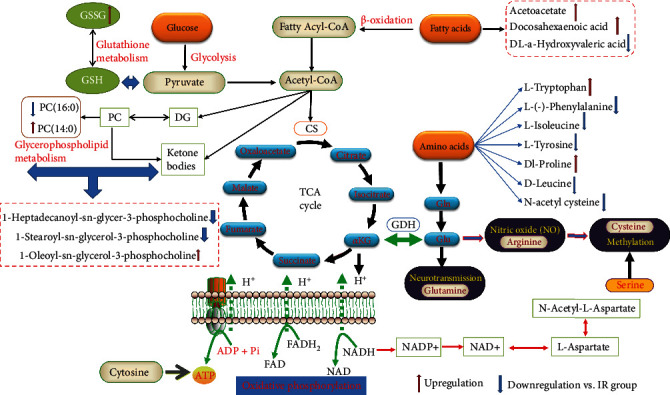
The metabolic pathway networks resulting from the cerebral IR under MJCPD intervention.

Our results demonstrated that MJCPD exerted the therapeutic effect against cerebral IR mainly through amino acid metabolisms including phenylalanine, tyrosine, and tryptophan metabolisms as well as valine, leucine, and isoleucine degradation. Previous studies have shown that amino acids were mainly involved in the pathological mechanisms of IS injury, especially excitatory amino acids, including glutamic acid and aspartic acid. When cerebral ischemia occurred, the release of excitatory amino acids increased, which easily led to excitatory amino acid toxicity [38]. Gamma aminobutyric acid (GABA) and glycine were generally considered inhibitory transmitters after cerebral ischemia, which could inhibit the excitation of neurons and reduce the damage of nerve cells caused by excitatory amino acids. After cerebral ischemia, the concentration of GABA increased sharply, which could reduce the damage caused by excessive release of glutamate [39]. N-Acetylaspartic acid was often regarded as a marker of neurons, and its metabolic disorder might trigger oxidative stress, which then led to neurodegenerative diseases. In the early stage of cerebral ischemia, the decrease in N-acetylaspartate mainly represented the decline of neuronal activity [40]. But so far, the role and function of tyrosine, tryptophan, and methionine were not clear in cerebral ischemia, which required further study to explore their specific mechanisms. In the meantime, studies have shown that serine and alanine could be converted into pyruvate through the process of glucose degradation, while isoleucine and aspartate could produce acetyl coenzyme A by the tricarboxylic acid cycle, which was the energy source for the synthesis of ATP [41]. Above all, the disorder of amino acid metabolism was involved in the whole process of cerebral ischemia. In our study, we found that there were 9 potential biomarkers that participated in amino acid metabolism; they were L-isoleucine, L-(-) phenylalanine, D-phenylalanine, L-tryptophan, L-tyrosine, DL-proline, D-leucine, N-acetyl cysteine, and pyroglutamic acid, as shown in Figure 11.

### 4.5. Mitochondrial Energy Metabolism

When cerebral infarction occurred, energy metabolism disturbance appeared in the whole brain, resulting in the interruption of mitochondrial metabolism and the reduction of ATP production, which led to the production of lactic acid through anaerobic degradation of glucose. Meanwhile, creatine phosphate, another way of energy storage, was also decomposed into creatine for energy supply [42]. Once severe cerebral ischemia occurred, the death of neurons and decrease in glucose metabolism appeared one after another, which indicated that energy metabolism disorder was the root cause of cerebral IR injury. However, in the brain area without severe ischemia, the glucose metabolism rate was enhanced and glucose anaerobic digestion as well as lactic acid level was increased, leading to mild and microacidosis to form limited ischemic protection. The abnormal levels of lactate and creatine would appear after IS; in particular, the increase in lactate was regarded as a sensitive indicator of early ischemia [43]. In our study, the concentration of lactic acid in the IR model group was enhanced as compared with SHAM rats, and expectedly, after being treated with MJCPD, the levels of acetoacetate and lactic acid were significantly decreased, which implied that energy metabolism was abnormal. At the same time, we found that acetylacetic acid was an intermediate product of fatty acid metabolism, which indicated that fatty acid *β*-oxidation was also an important energy metabolism in brain tissue besides the glucose anaerobic pathway [44]. The tricarboxylic acid cycle was the main way for the body to obtain energy; leucine and isoleucine could be converted into acetyl coenzyme A and entered the tricarboxylic acid cycle to provide energy [45]. These findings indicated that the occurrence of IR was related to the oxidative respiratory chain and production of energy, including fatty acid oxidation, glucose metabolism, and tricarboxylic acid cycle. Because the above-mentioned ways of energy generation were the necessary for maintaining human brain function, thus dysfunction may be resulting in energy production disorders and making the brain injury.

## 5. Conclusions

Our present study was set out to assess the beneficial effects and underlying mechanisms of MJCPD for the treatment of IS by the pharmacodynamics methods combined with the metabolomics techniques. The results explored that MJCPD possessed neuroprotective effects which mainly focused on the glucose, fatty acid, amino acid, glutamine, and phospholipid metabolisms, and all of which were related to the oxidative stress process.

## Figures and Tables

**Table 1:** The results of precision, stability, matrix effect, and recovery in positive mode.

*m*/*z*	RT (min)	Precision (RSD%)	Stability (RSD%)	Matrix effect (%)			Recovery (%)		
				High	Middle	Low	High	Middle	Low
132.0808	0.712	1.69	1.27	105.25	100.41	101.26	113.27	100.84	98.65
180.1019	1.474	13.54	14.15	105.9	94.02	110.99	101.45	110.07	97.63
205.0972	2.186	3.41	9.13	112.35	98.15	117.09	111.06	100.67	93.39
195.1016	3.313	1.57	6.21	110.97	98.45	96.02	103.71	97.61	96.11
223.0601	3.313	1.66	6.22	110.05	92.19	98.05	103.06	96.61	96.39
225.1485	7.057	11.35	6.08	102.38	99.53	102.81	98.41	86.56	102.84
469.3163	7.322	2.47	4.08	96.56	105.04	110.04	88.51	91.07	89.34
283.1693	14.429	3.82	4.39	104.82	104.62	106.21	95.83	100.46	104.16
285.2788	16.400	13.58	5.75	104.95	101.11	98.39	93.28	108.14	98.19
271.2632	18.272	7.59	4.47	100.27	95.32	99.75	106.88	101.08	104.97

**Table 2:** Potential biomarkers of cerebral ischemic reperfusion treatment with MJCPD in positive ion mode.

No.	*m*/*z*	RT (min)	Formula	Name	Adduct	IR vs. SHAM	MJCPDM vs.IR
1	132.1016	1.46	C_6_H_13_NO_2_	L-Isoleucine	M+H	↑^##^	↓
2	166.0861	1.69	C_9_H_11_NO_2_	L-(-)-Phenylalanine	M+H	↑^#^	↓
3	468.3079	7.32	C_22_H_46_NO_7_P	PC (14 : 0/0 : 0)	M+H	↓^#^	↑
4	166.0862	0.82	C_9_H_11_NO_2_	D-Phenylalanine	M+H	↑^#^	↓
5	149.0231	10.98	C_8_H_6_O_4_	5-Formylsalicylic acid	M+H_2_O-H	↑^#^	↓
6	510.3549	9.85	C_25_H_52_NO_7_P	1-Heptadecanoyl-sn-glycero-3-phosphocholine	M+H	↑^##^	↓^∗∗^
7	496.3391	8.58	C_24_H_50_NO_7_P	PC (16 : 0/0 : 0)	M+H	↑^#^	↓
8	132.0766	0.71	C_9_H_9_N	3-Methylindole	M+H	↑^##^	↓
9	331.284	14.23	C_19_H_38_O_4_	Glycerol 1-hexadecanoate	M+H	↑^##^	↓
10	205.0971	1.92	C_11_H_12_N_2_O_2_	L-Tryptophan	M+H	↓^##^	↑
11	182.081	1.43	C_9_H_11_NO_3_	L-Tyrosine	M+H	↑^##^	↓^∗∗^
12	112.0503	1.09	C_4_H_5_N_3_O	Cytosine	M+H	↑^#^	↑
13	522.3545	9.68	C_26_H_52_NO_7_P	1-Oleoyl-sn-glycero-3-phosphocholine	M+H	↓^###^	↑^∗∗∗^
14	116.0704	0.71	C_5_H_9_NO_2_	DL-Proline	M+H	↓^##^	↑^∗^
15	329.2474	14.09	C_22_H_32_O_2_	Docosahexaenoic acid	M+H	↑^##^	↑^∗^
16	524.371	0.83	C_26_H_54_NO_7_P	1-Stearoyl-sn-glycerol-3-phosphocholine	M+H	↑^##^	↓^∗∗^
17	132.1015	0.81	C_6_H_13_NO_2_	D-Leucine	M+H	↑^##^	↓
18	118.0648	2.16	C_5_H_10_O_3_	DL-a-Hydroxyvaleric acid	M+H	↑^#^	↓^∗^
19	162.0224	0.68	C_5_H_9_NO_3_S	N-Acetyl cysteine	M-H	↑^#^	↓^∗∗^
20	128.0425	0.62	C_5_H_7_NO_3_	Pyroglutamic acid	M+H	↑^#^	↓^∗∗^
21	117.0316	18.96	C_4_H_6_O_4_	Acetoacetate	M+H	↓^##^	↑^∗∗∗^

#*p* < 0.05, *^##^p* < 0.01, and *^###^p* < 0.001 as compared to the SHAM group; ^∗^*p* < 0.05, ^∗∗^*p* < 0.01, and ^∗∗∗^*p* < 0.001 as compared to the IR model group.

## Data Availability

The pharmacodynamics and metabolomics data used to support the findings of this study are included within the article.

## Abbreviations

CCA: Common carotid artery
ECA: External carotid
GABA: Gamma aminobutyric acid
GSH: Glutathione
GSSG: Glutathione disulfide
GSH-PX: Glutathione peroxidase
HE: Hematoxylin-eosin
HS: Hemorrhagic stroke
ICA: Internal carotid artery
IHC: Immunohistochemistry
IL: Interleukin
IR: Ischemia reperfusion
IS: Ischemic stroke
LDH: Lactate dehydrogenase
LDL: Low-density lipoprotein
L-LA: L-Lactic acid
MCAO: Middle cerebral artery occlusion
MDA: Malondialdehyde
MJCPD: Mi-Jian-Chang-Pu decoction
NO: Nitric oxide
PCA: Principal component analysis
PLS-DA: Partial least squares discriminant analysis
QC: Quality control
TCM: Traditional Chinese medicine
TNF: Tumor necrosis factor
t-PA: Tissue-type plasminogen activator
TTC: Triphenyltetrazolium chloride
VEGF-c: Vascular endothelial growth factor-c
GFAP: Glial fibrillary acidic protein.

## Supplementary Materials

Supplementary 1 Supplementary Figure 1: the total ion superposition diagram of the QC samples in positive ion mode.

Supplementary 2 Supplementary Figure 2: the representative total ion chromatogram of the sample in positive ion mode.
