# Supplementary figures and images for: Potential Therapeutic Effects of Mi-Jian-Chang-Pu Decoction on Neurochemical and Metabolic Changes of Cerebral Ischemia-Reperfusion Injury in Rats
Source: Oxid Med Cell Longev. 2022 May 6;2022:7319563. doi: 10.1155/2022/7319563 (PMC9107056; doi:10.1155/2022/7319563)

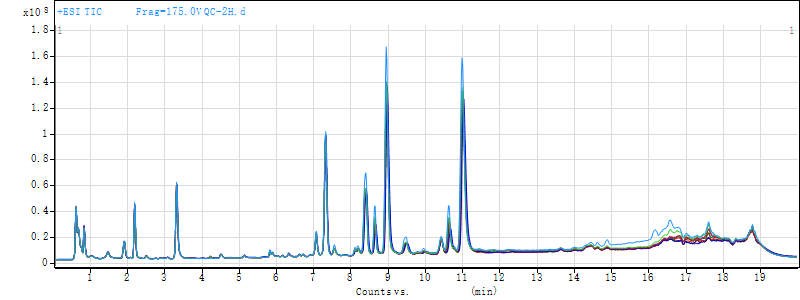


Supplementary Fig.1. The total ion superposition diagram of the QC samples in positive ion mode.

Supplement: Supplementary 1 — Supplementary Figure 1: the total ion superposition diagram of the QC samples in positive ion mode. [file 7319563.f1.docx]
