# Supplementary material for: Potential Therapeutic Effects of Mi-Jian-Chang-Pu Decoction on Neurochemical and Metabolic Changes of Cerebral Ischemia-Reperfusion Injury in Rats
Source: Oxid Med Cell Longev. 2022 May 6;2022:7319563. doi: 10.1155/2022/7319563 (PMC9107056; doi:10.1155/2022/7319563)
Supplement: Supplementary 2 — Supplementary Figure 2: the representative total ion chromatogram of the sample in positive ion mode. [file 7319563.f2.docx]

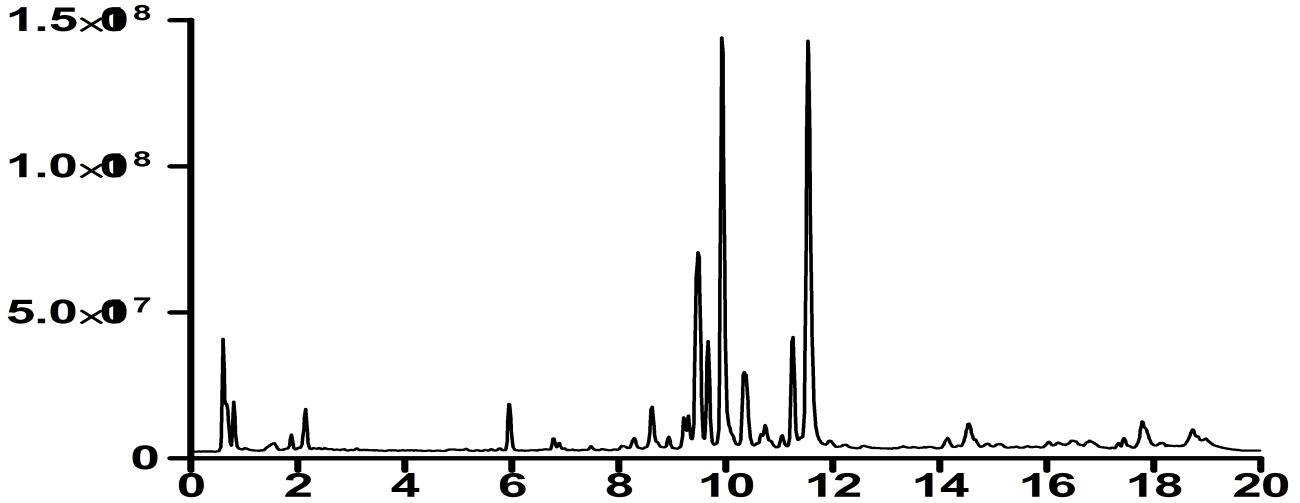


Supplementary Fig.2 The representative total ion chromatogram of the sample in positive ion mode.
